# The impact of mental illness on potentially preventable hospitalisations: a population-based cohort study

**DOI:** 10.1186/1471-244X-11-163

**Published:** 2011-10-10

**Authors:** Qun Mai, C D'Arcy J Holman, Frank M Sanfilippo, Jonathan D Emery

**Affiliations:** 1 School of Population Health, The University of Western Australia, 35 Stirling Highway, Crawley, WA, 6009, Australia; 2 School of Primary, Aboriginal and Rural Health Care, The University of Western Australia, 35 Stirling Highway, Crawley, WA, 6009, Australia

## Abstract

**Background:**

Emerging evidence indicates an association between mental illness and poor quality of physical health care. To test this, we compared mental health clients (MHCs) with non-MHCs on potentially preventable hospitalisations (PPHs) as an indicator of the quality of primary care received.

**Methods:**

Population-based retrospective cohort study of 139,208 MHCs and 294,180 matched non-MHCs in Western Australia from 1990 to 2006, using linked data from electoral roll registrations, mental health registry (MHR) records, hospital inpatient discharges and deaths. We used the electoral roll data as the sampling frame for both cohorts to enhance internal validity of the study, and the MHR to separate MHCs from non-MHCs. Rates of PPHs (overall and by PPH category and medical condition) were compared between MHCs, category of mental disorders and non-MHCs. Multivariate negative binomial regression analyses adjusted for socio-demographic factors, case mix and the year at the start of follow up due to dynamic nature of study cohorts.

**Results:**

PPHs accounted for more than 10% of all hospital admissions in MHCs, with diabetes and its complications, adverse drug events (ADEs), chronic obstructive pulmonary disease (COPD), convulsions and epilepsy, and congestive heart failure being the most common causes. Compared with non-MHCs, MHCs with any mental disorders were more likely to experience a PPH than non-MHCs (overall adjusted rate ratio (ARR) 2.06, 95% confidence interval (CI) 2.03-2.09). ARRs of PPHs were highest for convulsions and epilepsy, nutritional deficiencies, COPD and ADEs. The ARR of a PPH was highest in MHCs with alcohol/drug disorders, affective psychoses, other psychoses and schizophrenia.

**Conclusions:**

MHCs have a significantly higher rate of PPHs than non-MHCs. Improving primary and secondary prevention is warranted in MHCs, especially at the primary care level, despite there may be different thresholds for admission in people with established physical disease that is influenced by whether or not they have comorbid mental illness.

## Background

Health care disparities in vulnerable populations are a public health and ethical challenge [1]. Previous studies have been predominately focused on racial/ethnic [2], socioeconomic [3] or geographic related disparities [4]. Mental illness related disparities have been given less attention [5].

About 1 in 5 Australian adults has a clinically diagnosable mental illness [6]. This vulnerable group not only suffer from debilitating disability and a high risk of suicide [7], but also high risks of morbidity and mortality from physical illness [8]. The 2000 *Duty to Care *study found that Western Australian (WA) mental health clients (MHCs), generally with moderate to severe mental illness, had an overall 2.5 times higher mortality rate than the general population, mostly due to preventable physical diseases [8]. Apart from genetic, lifestyle, social and environmental factors, disparities in access to, and the quality of, physical health care may also contribute to this [9]. Access to care is a prerequisite for quality of care, whilst primary care is a foundation for population health, especially for vulnerable groups [10]. Our previous study found that MHCs had substantially more general practitioner (GP) visits than non-MHCs [11]. This suggests that, in Australia, with its universal health insurance cover provided by Medicare, it appears unlikely that limited access to primary care explains poor physical health outcomes in MHCs. We have therefore turned our focus on whether disparities exist in the quality of primary care using potentially preventable hospitalisations (PPHs) as an indicator [12]. This is because the data we have cannot measure quality of primary care directly but indicators of it. These indicators of quality of primary care are useful screening tools for potential problems in preventive and primary care, and that determining whether there is a quality problem requires more in-depth analysis.

PPH medical conditions, also known as avoidable hospitalisation conditions or ambulatory care sensitive conditions, are those for which good primary and preventive care are thought that could potentially prevent the need for hospitalisation, and are thus considered as indicators for access to, and the quality of, primary care [12]. Early studies categorised PPH medical conditions into: vaccine-preventable, chronic and acute [13]. Adverse events are now also included, adding a safety measure of quality [13].

Previous studies have examined: (i) mental illness-related disparities in the quality of physical health care across several physical conditions, such as coronary heart disease [14] and diabetes mellitus [15]; and (ii) racial/ethnic, socioeconomic and geographic-related disparities in PPHs [16-18]. However, to our knowledge, no study has specifically examined mental illness-related disparities in PPHs. To address this, we linked multiple population-based datasets to answer four specific questions: (i) do MHCs have more PPHs than non-MHCs; (ii) which PPH category/medical condition has the highest relative risk; (iii) do the associations vary by category of mental disorders; and (iv) what would be the potential savings in hospital admissions if MHCs had received the 'same' quality of primary care as non-MHCs.

## Methods

We conducted a population-based retrospective cohort study for the period 1 January 1990 to 30 June 2006 in WA.

### Data sources

We linked four de-identified routinely collected datasets from the WA Data Linkage System [19,20] (see Additional File 1, Table S1): (i) mental health registry (MHR, 8% of the general population), (ii) electoral roll registrations (86% of the general population aged ≥ 18 years), (iii) hospital inpatient discharges, and (iv) death registrations.

The MHR contained mental health inpatient data from all psychiatric institutions, public and private general hospitals, and outpatient data from public mental health clinics, community mental health services and psychiatric residential units. Data from private psychiatrists and GPs treating mental disorders were collected and administered by the Commonwealth Department of Health and thus not covered in the MHR.

### Study cohorts

**Figure 1:**
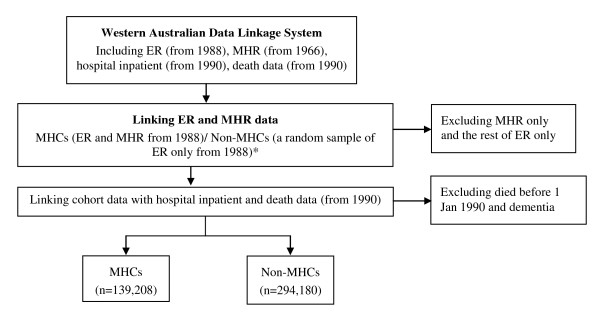
**Selection of study cohorts**. Abbreviations: ER = electoral roll registrations, MHCs = mental health clients, MHR = mental health registry. * Non-MHCs matched 2:1 with MHCs by 5-year age group, sex and being a current elector at study entry. The final ratio of non-MHC to MHC was 2.11:1 after excluding MHCs with dementia.

To enhance the internal validity of the study, we used the electoral roll as the sampling frame for both MHCs and non-MHCs to ensure that the baseline populations - MHCs and non-MHCs - came ultimately from the same source (Figure 1). The WA state-wide electoral roll data is a single dataset that contains the complete set of 1988 WA state electoral roll registrations and all quarterly updates of changes of enrolment status since then, e.g. new enrolments as well as removals from the roll due to unsound mind, moved out of state or death. The dataset contains information on encrypted ID, date of transaction, reason for the transaction, sex, date of birth and place of residence. MHCs were defined as people on the electoral roll, who were also on the MHR (about 80% of MHR) and still alive from 1 January 1990 onwards. Non-MHCs were a random sample of people who were on the electoral roll, but never recorded in the MHR. They were matched 2:1 with MHCs by 5-year age group, sex and current electoral roll registration at study entry. Age is calculated from date of birth and study entry date. For MHCs, the study entry date was 1 January 1990 for patients recorded in the MHR before 1 January 1990, or the first date of registration on the MHR for those recorded later. For non-MHCs, it was the same as that of their matched MHCs. The start of follow-up (T_0_) was the entry date for both cohorts.

### Variables and measurements

#### Outcome variables

The outcome measure was the rate of PPHs during the follow-up period from 1 Jan 1990 to 31 Dec 2006. There were four categories of PPH medical conditions investigated (see Additional File 1, Table S2): vaccine-preventable, chronic, acute and adverse drug events (ADEs). The first three categories were identified using the Australian Institute of Health and Welfare definition [21], with diabetes-related renal dialysis being counted only once for each person. This was because there were a large number of diabetes-related renal dialysis admissions, and for the purpose of this study we considered these as one episode of care per person and counted as one hospital discharge with a length of stay of one day. We substituted adverse events with ADEs as the majority of advents events were ADEs, defined as any adverse effect of drugs, medicines or biological substances in therapeutic use (ICD-9-CM, E930-E949 or ICD-10-AM, Y40-Y59). Follow-up time was censored at 30 June 2006 or an earlier date of death.

#### Exposure variables

We ascertained the principal mental health diagnosis for each MHC using a previously published method [11,22], which assigned patients to their most significant mental health diagnosis on an hierarchy of severity (see Additional File 1, Table S1). People with dementia (n = 15,764) were excluded due to their high use of residential care, but their matched non-MHCs were retained to maintain the high precision possible in a study of this size. The remaining records were then grouped into one of ten mutually exclusive categories of mental disorders (see Additional File 1, Table S1).

#### Potential confounders

Scores for social disadvantage and residential remoteness were derived from the Index of Relative Socio-Economic Disadvantage (IRSD) [23] and the Accessibility/Remoteness Index of Australia (ARIA) [24] based on place of residence at the Australian Census date. Social disadvantage scores were grouped into five levels (the lowest 10% of IRSD scores of the WA general population, 10% to < 25%, 25% to < 50%, 50% to < 75% and ≥ 75%) and remoteness scores were grouped into metropolitan, rural and remote. The category of the lowest 10% IRSD was created because a high proportion of MHCs fell into this group. Age, level of social disadvantage, level of residential remoteness and year at the start of follow up were measured at T_0_. Physical comorbidities were measured by the Charlson Index [25] based on inpatient data with a five-year look-back period from T_0_.

### Statistical analysis

We compared patient characteristics and crude numbers of hospital discharges, bed days and average length of stay during the entire follow-up period between MHCs and non-MHCs using bivariate analyses (chi-squared or unpaired tests for categorical variables, two-tailed *t- *or Mann-Whitney tests for continuous variables).

We then compared rates of PPHs between the two cohorts using unadjusted and adjusted negative binomial regression. Adjusted analyses controlled for potential patient-level confounders: 5-year age group, sex, Indigenous status, level of social disadvantage, level of residential remoteness, physical comorbidities (Charlson Index as a continuous variable) and year at T_0_. We repeated the above analyses for each PPH category and condition. Because MHCs represented a heterogeneous group, we repeated the above analyses, comparing MHCs in each category of mental disorder with non-MHCs.

We calculated the etiological fraction of mental illness attributable to disparities in PPHs as (Rate Ratio-1)/Rate Ratio and estimated the potential savings in hospital admissions and bed days if MHCs had experienced the same PPH outcomes as non-MHCs, determined by multiplying hospital discharges by the etiological fraction.

Missing values for each variable were treated as a separate exposure category so that all subjects were included in the multivariate analyses. Stata version 10.0 for Windows (StataCorp, College Station, Tex, USA) was used for all analyses.

The study was approved by the Human Research Ethics Committees of The University of Western Australia, and health departments of the Australian and WA governments.

## Results

### Patient characteristics

The study cohorts comprised 139,208 MHCs and 294,180 non-MHCs. Characteristics of MHCs and non-MHCs at T_0 _are shown in Table 1. Relative to non-MHCs, MHCs were more likely to be Indigenous, socially disadvantaged, living in rural or remote WA and have more physical comorbidities (all p-values < 0.001). The distribution of mental disorders among MHCs is also shown in Table 1, with affective psychoses and neurotic disorders being the most common.

**Table 1:** Characteristics of mental health clients (MHCs) and non-MHCs at the start of follow up

Characteristic*	MHCs (n = 139,208)	Non-MHC (n = 294,180)
Age, years, mean (SD)	43.7 (18.6)	45.1 (19.7)
Sex, % male	40.3%	40.4%
Indigenous status, %		
Indigenous (excluding missing)	5.7% (5.7%)	2.1% (2.3%)
Non-Indigenous (excluding missing)	93.9% (94.3%)	87.9% (97.7%)
Missing	0.4%	10.0%
Level of social disadvantage		
Most disadvantaged (the lowest 10% of IRSD scores**)	14.8%	10.9%
More disadvantaged (10% to < 25%)	18.7%	15.8%
Little disadvantaged (25% to < 50%)	25.4%	23.6%
Less disadvantaged (50% to < 75%)	18.5%	20.1%
Least disadvantaged (75%+)	22.6%	29.6%
Residential remoteness		
Metropolitan WA	68.6%	73.0%
Rural WA	22.7%	20.6%
Remote WA	8.7%	6.4%
Physical comorbidity (Charlson) score, mean (SD)	1.28 (2.34)	1.04 (2.24)
Category of mental disorders (%)		
Alcohol/drug disorders	8.0%	-
Schizophrenia	4.2%	-
Affective psychoses	17.1%	-
Other psychoses	6.7%	-
Neurotic disorders	16.2%	-
Personality disorders	2.5%	-
Adjustment disorders	7.3%	-
Depressive disorders	4.4%	-
Other mental disorders	8.0%	-
In MHR but had no mental health diagnosis, including suicide attempts	25.5%	-

Abbreviations: MHCs = mental health clients, SD = standard deviation, WA = Western Australia, MHR = mental health registry.

*P-values < 0.001 for all comparisons between MHCs and non-MHCs, except for sex (p = 0.52).

**The lowest 10% of IRSD scores of the Western Australian general population.

### Descriptive analyses

Numbers of hospital discharges, bed days and average length of stay of total hospital admissions and PPHs (total and by PPH category and medical condition) for both cohorts during entire follow-up period are shown in Table 2. PPHs accounted for more than 10% of all hospital discharges in both groups (Table 2). The most common PPH medical conditions in MHCs were diabetes and its complications, ADEs, chronic obstructive pulmonary disease (COPD), convulsions and epilepsy, and congestive heart failure. ADEs, diabetes and its complications, COPD and congestive heart failure had the highest numbers of total bed days. PPH medical conditions with the longest average length of stay were gangrene, nutritional deficiencies, ADEs, and influenza and pneumonia.

**Table 2:** Hospital discharges, bed days and average length of stay for potentially preventable hospitalisations by study cohort, PPH category and condition, 1 January 1990 to 30 June 2006

PPH category/condition	Discharges in MHCs (n = 139,208)	Discharges in Non-MHC (n = 294,180)	Bed-days in MHCs (n = 139,208)	Bed-days in Non-MHC (n = 294,180)	ALOS (days) in MHCs (n = 139,208)	ALOS (days) in Non-MHC (n = 294,180)
Vaccine-preventable*	3,055	3,205	38,465	35,819	12.6	11.2
Influenza and pneumonia	2,786	2,879	35,847	33,154	12.9	11.5
Other vaccine-preventable conditions	270	328	2,686	2,682	9.9	8.2
Chronic*	48,350	62,644	309,642	403,664	6.4	6.4
Asthma	6,483	4,337	31,435	21,496	4.8	5.0
Congestive heart failure	6,844	13,138	58,954	121,571	8.6	9.3
Diabetes complications†	17,823	23,837	115,418	149,139	6.5	6.3
COPD	9,703	10,466	87,385	93,766	9.0	9.0
Angina	6,292	8,680	19,157	28,029	3.0	3.2
Iron deficiency anaemia	2,466	4,469	5,936	10,503	2.4	2.4
Hypertension	1,497	1,598	7,731	7,214	5.2	4.5
Nutritional deficiencies	21	10	363	289	17.3	28.9
Rheumatic heart disease	304	521	2,519	4,262	8.3	8.2
Acute*	33,329	30,720	148,426	156,055	4.5	5.1
Dehydration and gastroenteritis	5,578	6,404	17,696	20,861	3.2	3.3
Pyelonephritis	6,005	6,408	35,220	40,422	5.9	6.3
Perforated/bleeding ulcer	1,090	1,737	8,000	13,040	7.3	7.5
Cellulitis	4,334	4,574	23,089	28,398	5.3	6.2
Pelvic inflammatory disease	1,622	1,366	4,922	3,908	3.0	2.9
Ear, nose and throat infections	1,989	1,810	6,460	6,038	3.2	3.3
Dental conditions	3,724	5,147	5,812	6,658	1.6	1.3
Appendicitis with generalised peritonitis	244	497	2,096	3,138	8.6	6.3
Convulsions and epilepsy	8,036	1,830	31,376	9,259	3.9	5.1
Gangrene	726	980	14,050	24,460	19.4	25.0
**Adverse drug events**	16,002	15,683	211,495	174,786	13.2	11.1
Total PPHs*	96,862	107,821	665,517	720,975	6.9	6.7
**Total hospital admissions**†	912,175	1,013,403	5,579,134	4,413,672	6.1	4.4

Abbreviations: MHCs = mental health clients, PPH = potentially preventable hospitalisations, COPD = chronic obstructive pulmonary disease, ALOS = average length of stay (days).

*There were overlaps in definitions for PPHs, therefore the total numbers for each PPH categories and total PPHs were not the same as the sum of individual PPHs. †During follow-up period, there were a total of 183,846 hospital separations for renal dialysis among 919 persons. For the purpose of this study, all renal dialysis admissions were counted as one hospital episode for each person with length of stay of one day. The same definition was also applied for diabetes-related renal dialysis.

### Rate of PPHs

**Figure 2:**
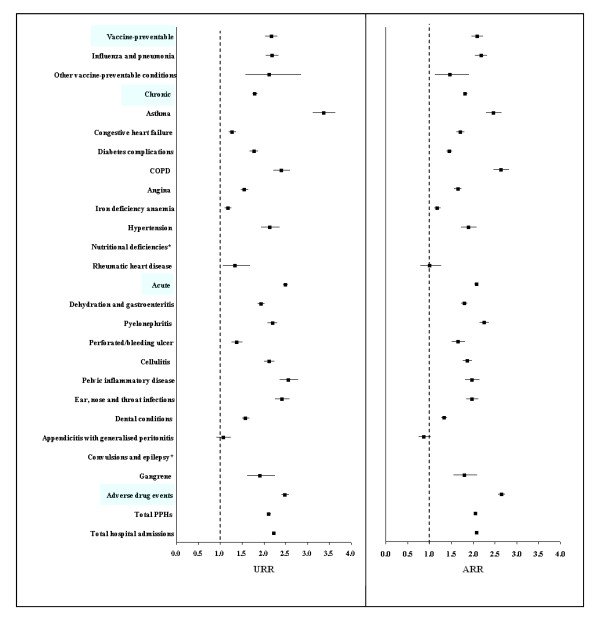
**Unadjusted (univariate analysis) and adjusted (multivariate analysis) rate ratios of potentially preventable hospitalisations (PPHs) from negative binomial regression analysis, stratified by PPH category and medical condition, 1 January 1990 to 30 June 2006**. Abbreviations: PPHs = potentially preventable hospitalisations, URR = unadjusted rate ratio, ARR = adjusted rate ratio, MHCs = mental health clients, COPD = chronic obstructive pulmonary disease. Notes: URRs and ARRs for nutritional deficiencies and convulsions and epilepsy were eliminated from the figure because of the larger numbers, which can be found from Table 2. Multivariate regression model adjusted for 5-year age group, sex, Indigenous status, level of social disadvantage, level of residential remoteness, physical comorbidities and year at the start of follow up. The reference group was non-MHCs.

Compared with non-MHCs, MHCs had significantly higher rates of PPHs from both univariate and multivariate analyses (unadjusted rate ratio (RR) 2.12, 95% CI 2.07-2.16; adjusted RR (ARR) 2.06, 2.03-2.09) (Figure 2).

### By PPH category and condition

When we stratified analyses by PPH category and specific condition, ARRs were greater than one for all PPH categories and medical conditions, except appendicitis with generalised peritonitis (0.88, 0.75-1.03) (Table 3). By PPH category, ARRs were highest for ADEs (2.66, 2.58-2.74), followed by vaccine-preventable (2.10, 1.97-2.23), acute (2.08, 2.04-2.13) and chronic conditions (1.82, 1.78-1.87). By PPH medical conditions, ARRs were highest for convulsions and epilepsy (6.45, 5.89-7.07), nutritional deficiencies (4.81, 1.43-16.21), COPD (2.64, 2.47-2.83) and asthma (2.47, 2.30-2.66); although nutritional deficiencies had the lowest absolute numbers (21 in MHCs and 10 in non-MHCs). Other findings included that ARR increased with the year at the start of follow up (ARR 1.02, 95% 1.01-1.02, for each increment in year), age, social disadvantage, residential remoteness and level of physical comorbidities. It was also greater in females than males and Indigenous people than non-Indigenous people.

**Table 3:** Unadjusted (univariate analysis) and adjusted (multivariate analysis) rate ratios of potentially preventable hospitalisations (PPHs) from negative binomial regression analysis, stratified by PPH category and medical condition, 1 January 1990 to 30 June 2006

PPH category/medical condition	URR (95% CI)	P value	ARR (95% CI)	P value
**Vaccine-preventable**	2.17 (2.04-2.32)	< 0.001	2.10(1.970-2.23)	< 0.001
Influenza and pneumonia	2.19 (2.05-2.33)	< 0.001	2.19 (2.05-2.33)	< 0.001
Other vaccine-preventable conditions	2.13 (1.58-2.86)	< 0.001	1.47 (1.14-1.91)	0.004
**Chronic**	1.80 (1.74-1.86)	< 0.001	1.82 (1.78-1.87)	< 0.001
Asthma	3.37 (3.13-3.64)	< 0.001	2.47 (2.30-2.66)	< 0.001
Congestive heart failure	1.28 (1.19-1.37)	< 0.001	1.71 (1.62-1.81)	< 0.001
Diabetes and its complications	1.77 (1.68-1.87)	< 0.001	1.47 (1.40-1.53)	< 0.001
COPD	2.40 (2.22-2.60)	< 0.001	2.64 (2.47-2.83)	< 0.001
Angina	1.56 (1.48-1.65)	< 0.001	1.66 (1.57-1.74)	< 0.001
Iron deficiency anaemia	1.18 (1.11-1.27)	< 0.001	1.19 (1.11-1.27)	< 0.001
Hypertension	2.15 (1.94-2.37)	< 0.001	1.90 (1.73-2.09)	< 0.001
Nutritional deficiencies*	5.52 (1.91-15.97)	0.002	4.81 (1.43-16.21)	0.011
Rheumatic heart disease	1.34 (1.06-1.69)	0.015	1.01 (0.80-1.28)	0.917
**Acute**	2.50 (2.44-2.56)	< 0.001	2.08 (2.04-2.13)	< 0.001
Dehydration and gastroenteritis	1.94 (1.86-2.02)	< 0.001	1.80 (1.73-1.88)	< 0.001
Pyelonephritis	2.20 (2.09-2.32)	< 0.001	2.26 (2.15-2.36)	< 0.001
Perforated/bleeding ulcer	1.39 (1.27-1.52)	< 0.001	1.67 (1.52-1.82)	< 0.001
Cellulitis	2.13 (2.01-2.25)	< 0.001	1.87 (1.77-1.98)	< 0.001
Pelvic inflammatory disease	2.57 (2.36-2.79)	< 0.001	1.98 (1.82-2.16)	< 0.001
Ear, nose and throat infections	2.42 (2.25-2.59)	< 0.001	1.99 (1.85-2.13)	< 0.001
Dental conditions	1.59 (1.51-1.67)	< 0.001	1.34 (1.27-1.41)	< 0.001
Appendicitis with generalised peritonitis	1.07 (0.92-1.25)	0.385	0.88 (0.75-1.03)	0.118
Convulsions and epilepsy	10.67 (9.69-11.75)	< 0.001	6.45 (5.89-7.07)	< 0.001
Gangrene	1.91 (1.62-2.26)	< 0.001	1.81 (1.56-2.10)	< 0.001
**Adverse drug events**	2.49 (2.41-2.58)	< 0.001	2.66 (2.58-2.74)	< 0.001
**Total PPHs**	2.12 (2.07-2.16)	< 0.001	2.06 (2.03-2.09)	< 0.001
**Total hospital admissions**	2.23 (2.21-2.25)	< 0.001	2.09 (2.08-2.11)	< 0.001

Abbreviations: PPHs = potentially preventable hospitalisations, URR = unadjusted rate ratio, ARR = adjusted rate ratio, COPD = chronic obstructive pulmonary disease.

Unadjusted and adjusted rate ratios of hospitalisation were the second highest for nutritional deficiencies, after convulsions and epilepsy, but there were only 21 cases in MHCs and 10 in non-MHCs during the entire follow up period. Multivariate regression model adjusted for 5-year age group, sex, Indigenous status, level of social disadvantage, level of residential remoteness, physical comorbidities and year at the start of follow up.

The reference group was the comparison cohort, non-MHCs.

### Rate ratios by category of mental disorders

**Figure 3:**
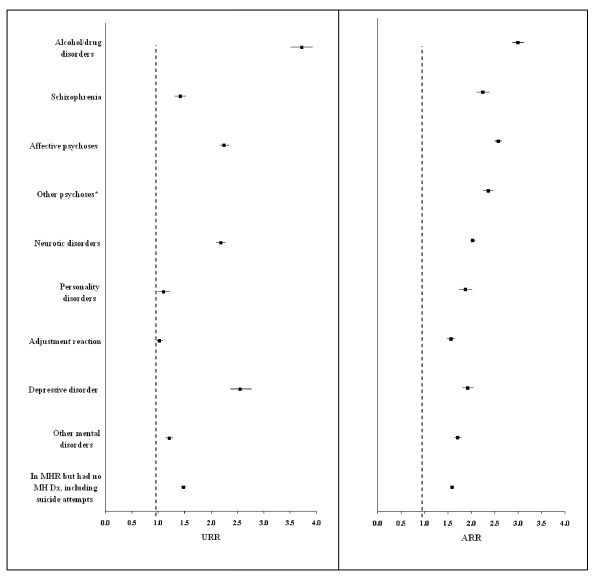
**Unadjusted (univariate analysis) and adjusted (multivariate analysis) rate ratios of total potentially preventable hospitalisations (PPHs) from negative binomial regression analysis, stratified by category of mental disorders**. Abbreviations: MHR = mental health registry, MH = mental health, Dx = diagnosis, URR = unadjusted rate ratio, ARR = adjusted rate ratio, MHCs = mental health clients. Note: URR for other psychoses was eliminated from the figure because of the large number (URR 6.42, 95% CI 6.02-6.85). The drop in other psychoses from an URR of 6.42 to an ARR of 2.47, mainly due to their older age and high level of physical comorbidities. Multivariate regression model adjusted for 5-year age group, sex, Indigenous status, level of social disadvantage, level of residential remoteness, physical comorbidities and year at the start of follow up. The reference group was non-MHCs.

ARRs of PPHs in MHCs with any mental disorders were all greater than one (Figure 3), with the highest for alcohol and drug disorders (3.00, 2.88-3.13), affective disorders (2.58, 2.50-2.66), other psychoses (2.36, 2.26-2.47) and schizophrenia (2.25, 2.12-2.39). The leading causes of excess PPHs for these four categories of mental disorders are shown in Table 4.

**Table 4:** Adjusted rate ratios of potentially preventable hospitalisations, by selective categories of mental disorders

Category of mental disorder and PPH	ARR	95% CI
**Alcohol and drug disorders**		
Nutritional deficiencies	19.45	(1.79-211.88)
Convulsions and epilepsy	16.35	(13.47-19.84)
Gangrene	5.22	(3.86-7.05)
COPD	4.49	(2.57-7.85)
Other vaccine-preventable conditions	4.47	(3.76-5.30)
Perforated/bleeding ulcer	4.21	(3.46-5.11)
**Affective disorders**		
Nutritional deficiencies	18.68	(2.49-140.27)
Convulsions and epilepsy	6.51	(5.59-7.59)
Adverse drug events	4.53	(4.31-4.78)
Asthma	2.98	(2.59-3.41)
COPD	2.98	(2.59-3.40)
**Other psychoses**		
Convulsions and epilepsy	19.63	(15.94-24.17)
Pyelonephritis	3.22	(2.89-3.58)
Adverse drug events	3.11	(2.89-3.36)
Other vaccine-preventable conditions	2.91	(1.33-6.38)
Gangrene	2.50	(1.78-3.51)
**Schizophrenia**		
Nutritional deficiencies	53.48	(1.97-1451.32)
Adverse drug events	6.91	(6.30-7.58)
Convulsions and epilepsy	5.97	(4.52-7.87)
Congestive heart failure	2.91	(2.34-3.62)
Influenza and pneumonia	2.63	(2.11-3.27)

Abbreviations: ARR = adjusted rate ratio, COPD = chronic obstructive pulmonary disease.

### Potential savings

Greenland and Robins have described three different types of attributable fraction in the exposed [26]. In this study, the incidence density fraction of PPHs in MHCs attributable to their mental illness was 51.4%, based on an ARR of 2.06 (Table 3). Strictly, the incidence density fraction will only equal the true etiologic fraction when exposure acts independently of background causes [27], but otherwise the approximation is often conservative [28]. Thus, a rough estimate of the potential savings is that if the elevated rate of PPHs in the mentally ill observed in Western Australia was caused by suboptimal health care, then had that inequality in care been redressed, it could have translated into 49,787 (i.e., 96,862 × 51.4%) fewer hospital admissions at the times that they occurred during follow-up.

## Discussion

We used a population-based approach to examine mental illness related disparities in PPHs. The results showed that: (i) diabetes and its complications, ADEs, COPD, convulsions and epilepsy, and congestive heart failure were the most common PPHs in MHCs; (ii) on average, MHCs were about twice as likely as non-MHCs to experience a PPH, with the largest differences occurring in PPHs for convulsions and epilepsy, nutritional deficiencies, ADEs, COPD and asthma; (iii) although ARRs were greater than 1 in MHCs with any mental disorders, it was higher in those with relatively more debilitating mental disorders such as alcohol and drug disorders, affective psychoses, other psychoses and schizophrenia; (iv) the disparities seem to have been increasing over the years; and (v) potentially half of all acute hospital admissions for PPHs could be avoided if MHCs had received preventive and primary care that achieved the same PPH outcomes as observed in non-MHCs.

The strengths of our study were: (i) use of validated population-based linked data with over 400,000 people in the study populations, (ii) inclusion of wide spectrums of mental disorders and PPH medical conditions, (iii) use of an internally valid comparison cohort of non-MHCs, and (iv) long-term follow-up (up to 16.5 years). It allows the scope of the problem to be quantified using innovative methods from well-established datasets with complete capture of population-wide data, so that changes in the situation can be monitored and the effectiveness of large-scale interventions and policy changes can be examined.

Limitations included firstly we did not have the data to directly measure the quality of preventive/primary care but an indicator of it. Thus, we cannot answer the question about whether higher rates of PPHs are due to poor quality of primary care or other factors. Nevertheless, the results show that there may be potential problems in preventive/primary care in MHCs that warrant more in-depth analysis. Secondly, the lack of data on ambulatory services provided by private psychiatrists and GPs treating mental disorders. This limited the extrapolation of our findings for all people with mental illness because some people with mental illness in Australia receive treatment only through these private sectors. Nevertheless, the MHR included about 40% of people with mental illness, generally with moderate to severe illness, whose physical health and physical health care disparities were probably greater than the remainder of people with mental illness. Moreover, we used the MHR for selection of our mental health cohort as did the previous *Duty to Care *study [8] and GP utilisation study [11], thus ensuring continuity and integrity of our investigations and findings. Thirdly, the domain restriction to the electoral roll, which enhanced the internal validity, possibly reduced external validity. Disparities may be greater in MHCs who are not registered to vote (20% of the MHR), presumably those younger than 18 years old, with severe mental illness, homeless and new migrants. Moreover, the MHR captured only 40% of patients with mental illness, thus our non-MHCs almost certainly included some people with mental illness. This may have resulted in an underestimation of the true difference between MHCs and non-MHCs. Fourthly, the lack of information on lifestyle risk factors (e.g., smoking and obesity) or detailed clinical information (e.g., severity of disease) limited our adjustment for these factors in the analyses.

The significance and interpretation of the study findings need to take into account both absolute and relative measures. PPHs with both higher absolute numbers and ARRs deserve special attention, such as, diabetes and its complications, ADEs, COPD, and convulsions and epilepsy. Compared with non-MHCs, MHCs are more likely to have a higher prevalence of underlying PPH medical conditions [29,30] therefore have higher risks of hospitalisations for PPH medical conditions. Adequate access to and good quality of preventive and primary care are thought to lower the risks of hospitalisations for PPH medical conditions [12]. Our previous study reported that MHCs visit GPs significantly more often than non-MHCs, suggesting that the differences in the quality of primary care rather than access to primary care may deserve further investigations.

Although access to ambulatory specialist care may also impact on the risk of PPHs, primary care, not specialist care, is the ideal setting for primary and secondary prevention of PPH medical conditions, especially in MHCs with multiple comorbidities [31]. This is attributable to the core features of primary care: first point of contact, continuity, comprehensiveness, coordination and its lower cost [10].

The greatest disparities in patients with alcohol and drug disorders warrant special attention, as other work suggests that they are unlikely to receive preventive care [32]. Studies on race-related health care disparities have suggested that patient-provider interactions may be a major contributor to the disparities, thus the interpersonal aspects of the patient-provider relationship may contribute to more pronounced disparities in patients with alcohol and drug disorders [5].

Schizophrenia and affective psychoses are severe mental disorders. These disorders are associated with a high prevalence of lifestyle risk factors (eg. smoking and obesity), comorbid physical diseases and alcohol and drug disorders, poly-pharmacy and their adverse effects [30]. These, together with functional disabilities of patients who may be under the care of multiple health care professionals, increase risks of PPHs, especially for diabetes, ADEs and COPD.

The combination of high physical health needs and a poor quality of physical health care received has been suggested as the hallmark of medically vulnerable populations, including people with mental illness. Studying PPHs is a way to quantify the scope of the problem and the scope for health gain. Our study suggests that mental illness-related disparities in physical disease burden are real and substantial and poor quality of primary care may be a contributor. However, some apparent PPHs may be appropriate in those with mental illness because the threshold for admission may need to be lower if someone has a co-morbid mental condition which limits their functional ability.

The observation that differences between levels of healthcare according to mental health status is getting worse over time is interesting. This may be partly due to the combination of: (i) the dramatic deinstitutionalisation movement of the mental health reform that transforms mental health services from an institution-based to community-based care model, and (ii) inadequate supportive services and funding for supporting this movement so that people with mental illness may be more likely to fall through the cracks.

Further research is needed to examine in-depth whether there is a quality problem in primary care and to understand the extent to which patient, provider and system factors contribute to the quality of primary care and its implications for the outcomes of care and interventions.

## Conclusions

MHCs have a significantly higher risk of PPHs than non-MHCs. They deserve special attention in research, policy development and clinical practice, with the focus on improving primary and secondary prevention, especially at the primary care level. This is despite the different thresholds for admission in people with established physical disease that is influenced by whether or not they have comorbid mental illness, which is encouraging.

## Abbreviations

ADE: Adverse Drug Events; ARIA: Accessibility/Remoteness Index of Australia; ARR: Adjusted Rate Ratio; CI: Confidence Interval; COPD: Chronic Obstructive Pulmonary Disease; GP: General Practitioner; ICD-9-CM: The International Classification of Diseases; 9^th ^revision; Clinical Modification; ICD-10-AM: International Classification of Disease - 10^th ^revision - Australian Modification; IRSD: Index of Relative Socio-Economic Disadvantage; MHCs: Mental Health Clients; MHR: Mental Health Registry; PPHs: Potentially Preventable Hospitalisations; RR: Rate Ratio; T_0 _= start of follow up; URR: Unadjusted Rate Ratio; WA: Western Australia.

## Competing interests

The authors declare that they have no competing interests.

## Authors' contributions

QM and CDJH participated in the conception and design of the overall study, and formulation of analysis plan. QM researched data and wrote the manuscript. CDJH and FMS reviewed and edited the manuscript and contributed to the discussion. JDE critically revised the manuscript for important intellectual content. All authors have read and approved the final manuscript.

## Pre-publication history

The pre-publication history for this paper can be accessed here:

http://www.biomedcentral.com/1471-244X/11/163/prepub

## Supplementary Material

Additional file 1 **Table S1 - Data sources and definitions**. Table S1 shows data sources used in this study and definitions for severity of mental illness and category of mental disorders. **Table S2 - ICD codes used for identifying potentially preventable hospitalisations**. Table S2 shows ICD codes used for identifying potentially preventable hospitalisations. Click here for file
