# Supplementary material for: The impact of mental illness on potentially preventable hospitalisations: a population-based cohort study
Source: BMC Psychiatry. 2011 Oct 10;11:163. doi: 10.1186/1471-244X-11-163 (PMC3201897; doi:10.1186/1471-244X-11-163)
Supplement: Additional file 1 — Table S1 - Data sources and definitions. Table S1 shows data sources used in this study and definitions for severity of mental illness and category of mental disorders. Table S2 - ICD codes used for identifying potentially preventable hospitalisations. Table S2 shows ICD codes used for identifying potentially preventable hospitalisations. [file 1471-244X-11-163-S1.DOC]

**Additional Files**

**Table S1 - Data sources and definitions**

| **A. Data sources** | | **Period** | **Records** | | **Persons** |
| --- | --- | --- | --- | --- | --- |
| Hospital inpatient data | | 1980-2006 | 4,922,939 | | 525,983 |
| Mental health registry data | | 1966-2006 | 10,511,171 | | 340,841 |
| Electoral roll registrations | | 1988-2006 | 782,161 | | 483,524 |
| Deaths | | 1990-2006 | 80,546 | | 80,546 |
|  | | | | | |
| **B. Severity of mental illness** | **Diagnosis** | | | **ICD-9 or ICD-9-CM code** | |
| Severe | Dementia, organic psychotic conditions, schizophrenia and affective psychosis | | | 290, 293-296 | |
| Moderate | Alcohol and drug psychoses, paranoid states, other non-organic psychoses, neurotic disorders, personality disorders, sexual deviations, alcohol and drug dependence, and childhood disorders | | | 291-292, 297-305, 313-315 | |
| Mild | Adjustment reaction, reaction to stress, depressive disorders NEC*, conduct disorders NEC, special syndromes NEC and mental retardation | | | 306-312, 317-319 | |
| Other | Other than mental or behavioural disorders | | | not in 290-319 | |
|  | | | |  | |
| **C. Category of mental disorders** | | | | **ICD-9 or ICD-9-CM code**† | |
| Alcohol/drug disorders | | | | 291, 292, 303-305 | |
| Schizophrenia | | | | 295 | |
| Affective psychoses | | | | 296 | |
| Other psychoses | | | | 293, 294, 297-299 | |
| Neurotic disorders | | | | 300 | |
| Personality disorders | | | | 301 | |
| Adjustment reaction | | | | 309 | |
| Depressive disorder | | | | 311 | |
| Other mental disorders | | | | 302, 306-308, 310, 312-319 | |
| In the MHR, but had no mental health diagnosis, including self harm‡ | | | | Other than 290-319 | |

* NEC: not elsewhere classified.

† ICD-9 = International Classification of Diseases, ninth revision; ICD-9-CM = International Classification of Diseases, ninth revision, Clinical Modification.

‡ MHR: Mental Health Registry.

**Table S**2 - ICD codes used for identifying potentially preventable hospitalisations (PPH)

| **PPH category/condition** | **ICD-9 or ICD-9-CM codes** | **ICD-10-AM codes** |
| --- | --- | --- |
| *Vaccine-preventable* |  |  |
| Influenza and pneumonia | 481, 482.2, 482.3, 482.9, 483, 487.0, 487.1, 487.8 in any diagnosis field, excludes cases with additional diagnosis of 282.6 (sickle-cell disorders) and people under 2 months | J10, J11, J13, J14, J15.3, J15.4, J15.7, J15.9, J16.8, J18.1, J18.8 in any diagnosis field, excludes cases with additional diagnosis of D57 (sickle-cell disorders) and people under 2 months |
| Other vaccine-preventable conditions | 032, 033, 037, 045, 055, 056, 070.3, 072, 320.0 in any diagnosis field | A35, A36, A37, A80, B05, B06, B16.1, B16.9, B18.0, B18.1, B26, G00.0, M01.4 in any diagnosis field |
| *Chronic* |  |  |
| Asthma | 493 as principal diagnosis only | J45, J46 as principal diagnosis only |
| Congestive heart failure | 402.01, 402.11, 402.91, 428, 518.4 as principal diagnosis only, exclude cases with the following procedure codes: 35, 36, 37.5, 37.6, 37.7, 37.8 | I50, I11.0, J81 as principal diagnosis only, exclude cases with the following procedure codes: 33172-00, 35304-00, 35305-00, 35310-02, 35310-00, 38281-11, 38281-07, 38278-01, 38278-00, 38281-02, 38281-01, 38281-00, 38256-00, 38278-03, 38284-00, 38284-02, |
| Diabetes complications | 250 as principal diagnosis | E10–E14.9 as principal diagnoses |
|  | and 250 as additional diagnoses where the principal diagnosis was: | and E10–E14.9 as additional diagnoses where the principal diagnosis was: |
|  | Hypersmolarity (276.0) | Hypersmolarity (E87.0) |
|  | Acidosis (276.2) | Acidosis (E87.2) |
|  | Transient ischaemic attack (435) | Transient ischaemic attack (G45) |
|  | Nerve disorders and neuropathies (350-357) | Nerve disorders and neuropathies (G50–G64) |
|  | Cataracts and lens disorders (366, 379.3) | Cataracts and lens disorders (H25–H28) |
|  | Retinal disorders (361-363) | Retinal disorders (H30–H36) |
|  | Glaucoma (365) | Glaucoma (H40–H42) |
|  | Myocardial infarction (410) | Myocardial infarction (I21–I22) |
|  | Other coronary heart diseases (411-414) | Other coronary heart diseases (I20, I23–I25) |
|  | Congestive heart failure (428) | Congestive heart failure (I50) |
|  | Stroke and sequelae (430-432, 436, 438) | Stroke and sequelae (I60–I64, I69.0–I69.4) |
|  | Peripheral vascular disease (440-449) | Peripheral vascular disease (I70–I74) |
|  | Gingivitis and periodontal disease (523) | Gingivitis and periodontal disease (K05) |
|  | Kidney diseases (580-589, 590-599) | Kidney diseases (N00–N29) [including end-stage renal disease |
|  | Renal dialysis (V56) | Renal dialysis (Z49) |
| Chronic obstructive pulmonary disease | 466.0, 491, 492, 494, 496 as principal diagnosis only, 466.0 only with additional diagnoses of 491, 492, 494, 496 | J20, J41, J42, J43, J44, J47 as principal diagnosis only, J20 only with additional diagnoses of J41, J42, J43,J44, J47 |
| Angina | 411.1, 411.8, 413 as principal diagnosis only, exclude cases with procedure codes not in 0-86.99 | I20, I24.0, I24.8, I24.9 as principal diagnosis only, exclude cases with procedure codes not in blocks [1820] to [2016] |
| Iron deficiency anaemia | 280.1, 280.8, 280.9 as principal diagnosis only. | D50.1, D50.8, D50.9 as principal diagnosis only. |
| Hypertension | 401.0, 401.9, 402.00, 402.10, 402.90 as principal diagnosis only, exclude cases with procedure codes according to the list of procedures excluded from the Congestive cardiac failure category above. | I10, I11.9 as principal diagnosis only, exclude cases with procedure codes according to the list of procedures excluded from the Congestive cardiac failure category above. |
| Nutritional deficiencies | 260, 261, 262, 268.0, 268.1 as principal diagnosis only. | E40, E41, E42, E43, E55.0, E64.3 as principal diagnosis only. |
| Rheumatic heart disease | 390 to 399 as principal diagnosis only. | I00 to I09 as principal diagnosis only. (Note: includes acute rheumatic fever) |
| *Acute* |  |  |
| Dehydration and gastroenteritis | 276.5, 558.9 as principal diagnosis only. | E86, K52.2, K52.8, K52.9 as principal diagnosis only. A09.9 as principal diagnosis (aged >15 years only). |
| Pyelonephritis | 590.0, 590.1, 590.8, 599.0 as principal diagnosis only. | N10, N11, N12, N13.6, N39.0 as principal diagnosis only. |
| Perforated/bleeding ulcer | 531.0-531.2, 531.4-531.6, 532.0-532.2, 532.4-532.6, 533.0-533.2, 533.4-533.6, 534.0-534.2, 534.4-534.6 as principal diagnosis only. | K25.0, K25.1, K25.2, K25.4, K25.5, K25.6, K26.0, K26.1, K26.2, K26.4, K26.5, K26.6, K27.0, K27.1, K27.2, K27.4, K27.5, K27.6, K28.0, K28.1, K28.2, K28.4, K28.5, K28.6 as principal diagnosis only. |
| Cellulitis | 681, 682, 683, 686 as principal diagnosis only, exclude cases with procedure codes 01 to 86.99 except 86.0 where it is the only listed procedure. | L03, L04, L08, L88, L98.0, L98.3 as principal diagnosis only, exclude cases with any procedure in blocks 1820 to 2016, except if procedure is 30216-02, 30676-00, 30223-02, 30064-00, 34527-01, 34527-00, 90661-00 and this is the only listed procedure. |
| Pelvic inflammatory disease | 614 as principal diagnosis only. | N70, N73, N74 as principal diagnosis only. |
| Ear, nose and throat infections | 382, 462, 463, 465, 472.1 as principal diagnosis only. | H66, H67, J02, J03, J06, J31.2 as principal diagnosis only. |
| Dental conditions | 521, 522, 523, 525, 528 as principal diagnosis only. | K02, K03, K04, K05, K06, K08, K09.8, K09.9, K12, K13 as principal diagnosis only. |
| Appendicitis with generalised peritonitis | 540.0 in any diagnosis field | K35.0 in any diagnosis field |
| Convulsions and epilepsy | 345, 642.6, 780.3 as principal diagnosis only | G40, G41, O15, R56 as principal diagnosis only |
| Gangrene | 785.4 in any diagnosis field | R02 in any diagnosis field |
| *Adverse drug events* | E930-E949 | Y40-Y59 |
